# Distinct Binding Interactions of *α*_5_*β*_1_-Integrin and Proteoglycans with Fibronectin

**DOI:** 10.1016/j.bpj.2019.07.002

**Published:** 2019-07-05

**Authors:** Thomas M. Kennelly, Yiran Li, Yi Cao, Eva E. Qwarnstrom, Mark Geoghegan

**Affiliations:** 1 Department of Physics and Astronomy, University of Sheffield, Sheffield, United Kingdom; 2 Department of Infection, Immunity and Cardiovascular Disease, University of Sheffield, Sheffield, United Kingdom; 3 Department of Physics, Nanjing University, Nanjing, People’s Republic of China

## Abstract

Dynamic single-molecule force spectroscopy was performed to monitor the unbinding of fibronectin with the proteoglycans syndecan-4 (SDC4) and decorin and to compare this with the unbinding characteristics of *α*_5_*β*_1_-integrin. A single energy barrier was sufficient to describe the unbinding of both SDC4 and decorin from fibronectin, whereas two barriers were observed for the dissociation of *α*_5_*β*_1_-integrin from fibronectin. The outer (high-affinity) barriers in the interactions of fibronectin with *α*_5_*β*_1_-integrin and SDC4 are characterized by larger barrier heights and widths and slower dissociation rates than those of the inner (low-affinity) barriers in the interactions of fibronectin with *α*_5_*β*_1_-integrin and decorin. These results indicate that SDC4 and (ultimately) *α*_5_*β*_1_-integrin have the ability to withstand deformation in their interactions with fibronectin, whereas the decorin-fibronectin interaction is considerably more brittle.

## Significance

Dynamic single-molecule force spectroscopy was used to characterize the binding of two functionally distinct proteoglycans (PGs) (syndecan-4 and decorin) to the extracellular matrix protein, fibronectin, and to compare their binding characteristics with those of *α*_5_*β*_1_-integrin. The study demonstrates that PG binding is low affinity and exhibits a single barrier, in contrast to the double barrier representing *α*_5_*β*_1_-integrin binding, reflecting two interaction sites. Furthermore, although the energies of adhesion of the PGs are similar, their bonds with fibronectin are significantly different. Decorin exhibits a brittle bond, whereas the interaction with syndecan-4 is elastic. The distinct binding characteristics of the PGs, and the marked differences between their interaction with fibronectin and the *α*_5_*β*_1_-integrin binding, reflect specific molecular and biological features.

## Introduction

It is well known that the binding between ligands and receptors at the cell surface regulate cell function and behavior (1). In addition, cell responses are controlled by the cell environment such as the extracellular matrix (ECM) and binding of specific cell surface molecules to matrix proteins.

Fibronectin is a large protein of ∼0.5 MDa, which comprises two similar subunits attached through disulfide linkages. It is a primary ECM component and interacts with cell surface integrins and proteoglycans (2, 3).

Integrins are transmembrane proteins that comprise an *α*- and a *β*-subunit and provide attachment to the ECM and control responses to mechanical stimuli (4, 5, 6). The transmembrane domain is linked to the actin cytoskeleton via membrane proximal proteins, including talin and vinculin, and exists in high- or low-affinity states depending on their internal structure (7).

Members of the proteoglycan (PG) family are structurally characterized by highly sulfated glycosaminoglycan (GAG) chains anchored to a protein core. Many are cell surface components and exhibit coreceptor functionality for various systems (7, 8, 9, 10, 11, 12, 13, 14, 15). This includes heparan sulfate (HS) PGs (HSPGs; PGs containing HS GAG chains), which associate with regulatory receptor complexes to control signal amplification.

Syndecan-4 (SDC4; an ∼20 kDa HSPG and the smallest member of the syndecan family of biomolecules) regulates the fibroblast growth factor receptor function and so has significant effects in cellular development and proliferation (8, 16). SDC4 is constitutively expressed at focal adhesions, where it can form bonds with specific heparin-binding sites within the fibronectin core protein (3, 17). Decorin is a small, leucine-rich PG, ∼90–130 kDa in size, comprising a 42-kDa core protein to which a single chondroitin or dermatan sulfate GAG chain is attached at the N-terminus (18). Unlike SDC4, which is a membrane-spanning PG, decorin resides in the ECM and binds fibronectin and other ECM proteins such as collagen (19, 20, 21, 22). It has specific relevance in controlling regulatory events related to cell growth, morphogenesis, and immunity (23).

The distinct molecular features of integrins and PGs suggest that their control of cellular responses is underpinned in part by specific physical characteristics of their binding to the ECM. The impact of force and elasticity, generated through interactions with the ECM, on cell responses is well established (24, 25).

Atomic force microscopy (AFM) is an established technique for studying the binding between biomolecules (26, 27, 28). In addition to revealing dissociation rates, the AFM can provide information of the character of the bond under external stress, which, in the case of cell-ECM interactions and tissues under flow, may be physiologically relevant. The ability of the AFM to resolve these properties of bound systems stems from its capability in resolving the positions of individual energy barriers that govern unbinding events under different loading conditions (29).

In this work, the force of the ECM binding of PGs and *α*_5_*β*_1_-integrin is compared. Furthermore, the elastic character of both interactions is measured by extracting the width, *χ*_B_, of the energy barriers and determining the thermodynamic energy of adhesion, *ΔG*, for each from the dissociation rates.

**Figure 1:**
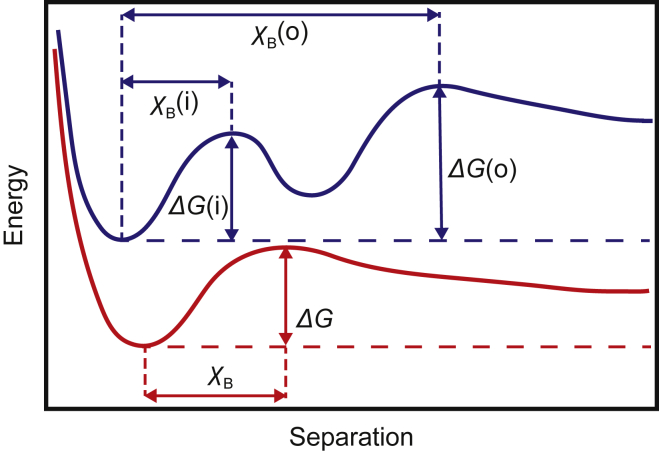
A schematic diagram of energy landscapes for protein unbinding. Two energy minima (*upper curve*) were observed for the fibronectin–*α*_5_*β*_1_-integrin interaction, but only one minimum (*lower curve*) was observed for fibronectin with the two PGs studied. *ΔG* represents the height of a barrier of width *χ*_B_. Here, (i) and (o) indicate the inner and outer barriers. Both curves have the same energy at large separations, so they are shifted for clarity. To see this figure in color, go online

Dynamic single-molecule force spectroscopy (DSMFS) was used to identify the energy barriers pertaining to the dissociation of the low molar mass PGs SDC4 and decorin with fibronectin (a schematic diagram is shown in Fig. 1). This study also extends this technique to compare the bond between PGs and fibronectin with energetic barriers describing the unbinding of *α*_5_*β*_1_-integrin. The interaction of fibronectin with *α*_5_*β*_1_-integrin is known to involve two energy barriers (30, 31), as is also schematized in Fig. 1. Force spectroscopy experiments have previously been used to characterize binding between fibronectin and heparin (32), and these are compared with the current results. It is shown that the energy barrier but not the dissociation rate constant is similar for SDC4 and heparin.

## Materials and Methods

Recombinant human decorin, recombinant human *α*_5_*β*_1_-integrin, and recombinant human SDC4 were purchased from R&D Systems (Minneapolis, MN). Bovine plasma fibronectin was purchased from Thermo Fisher Scientific (Waltham, MD). HS was purchased from Iduron (Manchester, UK). Hydrogen peroxide, sulfuric acid, (3-aminopropyl)triethoxysilane (APTES), and N-hydroxysuccinimide-poly(ethylene glycol)-maleimide (NHS-PEG-Mal) were purchased from Sigma-Aldrich (St Louis, MO).

Before substrates or probes were functionalized, they were cleaned using isopropanol, followed by 5 min in an oxygen plasma, and finally with deionized water.

### Functionalization of AFM tip

The PGs and integrin were attached to the AFM tip using short poly(ethylene glycol) (PEG]) units as linkers (33). Without these PEG chains, the PGs or integrin can irreversibly adsorb onto the AFM tips, making meaningful measurements impossible. Therefore, in a first step, the functionalized PEG is attached to an APTES-coated AFM tip. After this, it is possible to attach integrin or a PG using a standard chemical route.

The PGs were tethered via cysteine residues using silane chemistry (34). Silicon nitride microlever cantilever AFM probes were immersed in piranha solution (70% sulfuric acid and 30% hydrogen peroxide) for 30 min to hydroxylate the surface. A reactive amine group was then introduced by immersion in 3 *μ*L/mL APTES solution in toluene for 2 h and subsequently heated at ∼80°C in an oven for 30 min to stabilize the structure (35).

A PEG-linking region was attached to the tip by immersion in 0.5 mg/mL NHS-PEG-Mal in water for 2 h. The NHS terminus of the NHS-PEG-Mal binds with the exposed amine group on the tip resulting in a PEG-linked terminal maleimide (33). This Mal was bound directly to cysteines in the proteins of interest (SDC4, decorin, and *α*_5_*β*_1_ integrin) in 2 *μ*g/mL phosphate-buffered saline (PBS) solution (pH 7.4) at 2°C overnight (36). Between each step of the modification process, tips were dried using absorbent tissue and washed with the solvent required for the next step before being tissue dried again.

### Surface immobilization of fibronectin

A solution concentration of 20 *μ*g/mL fibronectin in PBS was selected and was applied to a gold surface overnight (12 mm diameter Pelco gold-coated AFM/STM metal specimen disks; Ted Pella, Redding, CA) at 4°C to ensure a complete coating. This concentration is consistent with other studies in which binding sites in fibronectin, such as the Arg-Gly-Asp (RGD) peptide, have been shown to be functional (30, 37, 38). Fibronectin readily adsorbs to gold, and no modification of protein or surface was necessary. A complete surface coating was verified using tapping mode AFM in a liquid PBS environment with an unmodified probe.

### Dynamic single-molecuie force spectroscopy

Before functionalization, the spring constant of the AFM tips was measured using the thermal method (39). The spring constant was typically 18 ± 2 pN nm^−1^.

**Figure 2:**
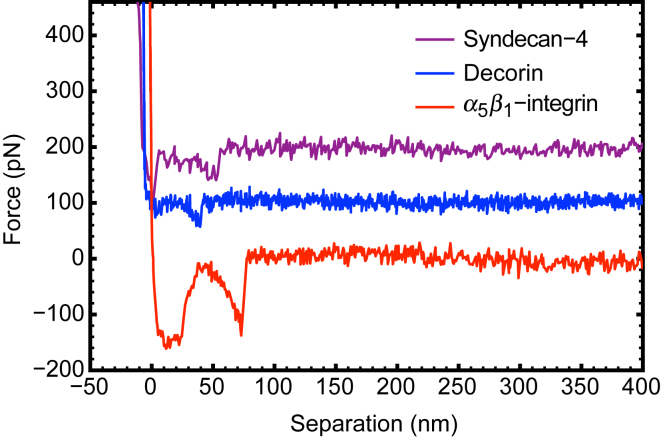
Representative force spectroscopy data for the unbinding of SDC4 (*top*), decorin (*middle*), and *α*_5_*β*_1_-integrin (*bottom*) from a fibronectin-immobilized surface. The retraction speeds for these data are 2, 1, and 3 *μ*m/s, respectively. For clarity, the data for SDC4 and decorin are offset by 200 and 100 pN, respectively. To see this figure in color, go online

DSMFS measurements were conducted on the unbinding of SDC4, decorin, and *α*_5_*β*_1_-integrin from fibronectin in PBS to identify characteristics of these distinct interactions. Force spectroscopy profiles are shown in Fig. 2. In DSMFS, the tip approaches the surface until contact, at which point the tip cannot move any further. The force on it increases until it reaches a set value known as the trigger force. The force is then held constant for a set period (dwell time) before the tip is retracted.

Control tests compared an SDC4-exhibiting probe interacting with fibronectin-immobilized or gold-coated surfaces and were conducted at a retraction speed of 1 *μ*m s^−1^ with a trigger force of 500 pN and a dwell time of 200 ms to enhance the probability of ligand-receptor binding. Longer dwell times were not selected because fibronectin has been shown to exhibit significant binding to Si_3_N_4_ cantilevers at exposure times of 1 s or longer (40). All control tests were conducted at 10 different regions on the fibronectin-coated substrate (the position was changed every 100 curves until 1000 curves were obtained) to ensure that measurements were not taken on unfunctionalized or otherwise adversely affected regions.

For the measurements of unbinding, the retraction speed was varied to control the loading rate, which is the product of the retraction speed and gradient of the rupture curve close to dissociation (32). Because this gradient is dependent on the cantilever and the polymers, including the bond, it is not possible to obtain specific loading rates for direct comparison of the different bonds. The 500-pN trigger force was retained from the control experiments. After the trigger force was achieved, the AFM tip was immediately retracted (no dwell). The position at which the measurement was taken was changed after 100 curves.

Hydrodynamic drag is a factor in experiments such as these, and a correction factor can be obtained from the gradient of a plot of half the difference between approach and retraction forces at large separations as a function of retraction speed. The fit for the cantilever used here reveals a drag coefficient of 3.6 pN s *μ*m^−1^, which is of the same order as those obtained in similar experiments (30, 32, 41).

### Analysis of unbinding events

Unbinding can be treated by the Bell-Evans model, which is a kinetic process of the escape from a potential under the influence of an external loading force (29, 42). It is possible to extract characteristics describing dissociation events between molecules by obtaining a linear fit between the rupture force and the natural logarithm of the loading rate for each contributing energy barrier. These include the dissociation rate, the thermodynamic energy of adhesion, and the barrier width (43). The relation between rupture force, *F*, and the loading rate, *r* (force per unit time), of the AFM cantilever is given by

(1) $$F=\frac{k_{\text{B}}T}{\chi_{\text{B}}}\ln\left(r\right)+\frac{k_{\text{B}}T}{\chi_{\text{B}}}\ln\left(\frac{\chi_{\text{B}}}{K_{\text{d}}k_{\text{B}}T}\right),$$

in which *k*_B_ is the Boltzmann constant, *T* is the absolute temperature, *χ*_B_ is the width (Fig. 1) of the energy barrier, and *K*_d_ is the dissociation (escape) rate. It is this dissociation rate that contains the thermodynamic energy of adhesion, *ΔG*, between the molecular bonds at the surface, given by the Arrhenius relation

(2) $$K_{\text{d}}=f_{\text{m}}\;\exp\left(\frac{-\Delta G}{k_{\text{B}}T}\right),$$

where *f*_m_ is the equilibrium dissociation rate, i.e., the dissociation rate when there are no external forces applied. For large or complex proteins, this rate is taken as *f*_m_ ≈ 10^7^ s^−1^ (44, 45, 46), which would be appropriate for simple interactions involving fibronectin. This value may still be an underestimate for SDC4 and decorin, although any uncertainty in *ΔG* is mitigated by its logarithmic dependency on *f*_m_. In general, an order of magnitude increase in *f*_m_ requires a corresponding 2.3*k*_B_*T* decrease in *ΔG*. The barrier width is not dependent on *f*_m_. A reduction in the height of the energy barrier is induced by the external force imparted on the bond and is assumed to increase linearly with the time under stress (47). The observed rupture force has then been shown to vary with the loading rate, which is dependent on the velocity of retraction of the tip from the sample.

## Results

### Control experiments

Control experiments were carried out on an uncoated surface to test that the chemically modified probes successfully exhibited proteins at cysteine residues via PEG chains. This is particularly important for syndecan, which is relatively depleted in cysteine compared to other PGs (48, 49). In the absence of thiols, nonspecific maleimide binding with amines is also possible (50), although this is a slow reaction, increasing with pH (51). The first control involved recording the number of events in 1000 curves on a fibronectin-coated substrate and an SDC4-exhibiting probe in a liquid environment of PBS. This surface was then replaced with an unmodified gold substrate, which was immersed in new PBS, and a further 1000 curves were taken. Single-molecule events characterized in the measurements between the SDC4 probe and the fibronectin surface were not observed on an uncoated gold substrate, indicating that the rupture events are due to the functionalized probe and fibronectin.

HS is generally found as a PG component but is structurally almost identical to isolated GAG heparin. In fact, they differ only in the degree of sulfation along their chains; HS contains various sulfated domains interspaced with nonsulfated domains, whereas heparin is uniformly sulfated along its length. To test for nonspecific binding, control experiments were performed for an SDC4 probe on a fibronectin surface in which heparin-binding sites were blocked by immersing the surface in free HS for 30 min. These experiments identified a low level of nonspecific binding that corresponded to ∼10% of events. This agrees with published data and demonstrates that DSMFS is a reliable tool for measuring these interactions (52).

### Unbinding of *α*_5_*β*_1_-integrin and fibronectin

**Figure 3:**
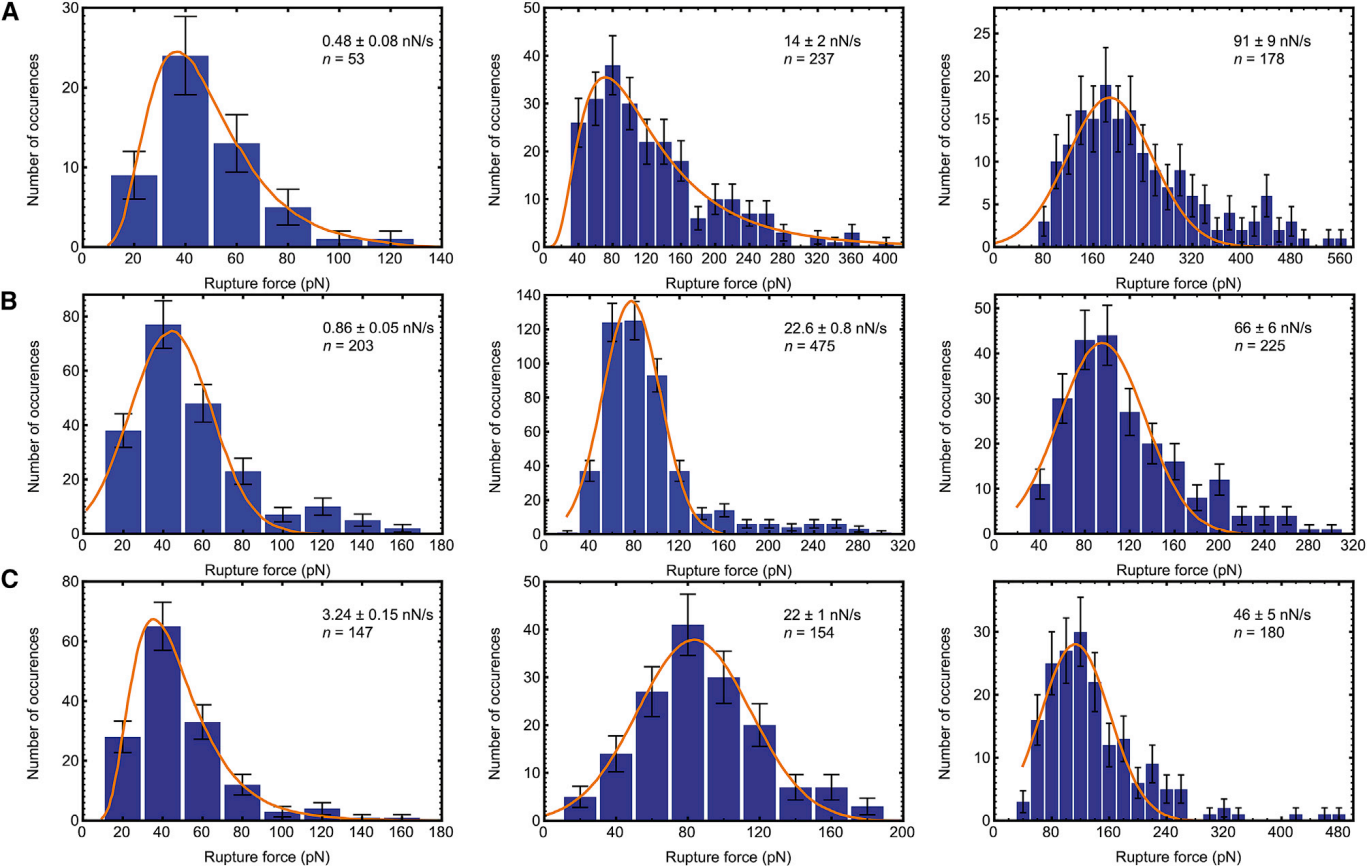
Frequency distributions for extracted rupture forces, taken at different velocities for the unbinding of fibronectin from *α*_5_*β*_1_-integrin (*A*), SDC4 (*B*), and decorin (*C*). The number of measurements, *n*, from which the distributions were obtained, is shown in each panel. The fits (shown) are to either Gaussian or log-Gaussian models, and the modal averages of the rupture force for the corresponding loading rate were obtained. (These may be distinguished by noting that the fit to a log-Gaussian model passes through the origin.) Included errors correspond to the width of the 95% confidence interval for the fit. Standard error values for each fit, provided by statistical software package GraphPad, were significantly smaller than the error values (95% confidence interval width) quoted here. The log-Gaussian fit was used when the Gaussian fit was unsatisfactory, although the modal average is model independent. To see this figure in color, go online.

**Figure 4:**
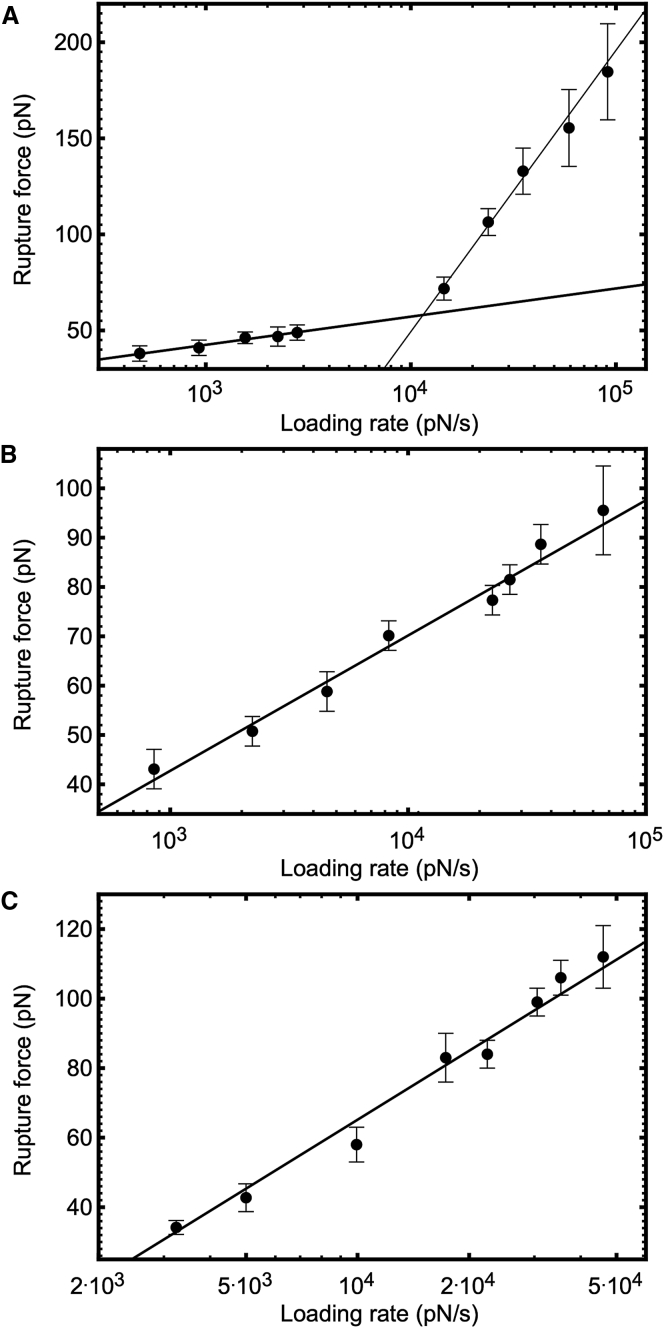
The dynamic force spectrum describing the unbinding between fibronectin and *α*_5_*β*_1_-integrin (*A*), SDC4 (*B*), and decorin (*C*). The linear increase of the average rupture force with the logarithm of the average loading rate for each pulling velocity, fitted using the Bell-Evans relation (Eq. 1), is shown. Included errors correspond to the width of the 95% confidence interval from which average rupture forces were extracted. Two energy barriers were observed for the unbinding of fibronectin from *α*_5_*β*_1_-integrin.

Force pulling of an *α*_5_*β*_1_-integrin–exhibiting probe from a fibronectin-immobilized surface were conducted over a range of loading rates between 0.5 and 91 nN s^−1^. The distribution of rupture forces is shown in Fig. 3 *A*, and the dynamic force spectrum describing the unbinding is presented in Fig. 4 *A*. The energy landscape in this range is governed by two energy barriers. When fitted with the Bell-Evans model in Eq. 1, the low-affinity (inner) barrier is described by a linear regression of

(3) F=(60±3)lnr–(500±30),

with a correlation factor of R^2^ = 0.9935. The high-affinity (outer) barrier is described by

(4) F=(6.2±0.6)lnr–(1±4),

with a correlation factor of R^2^ = 0.9712. The rupture force is in pN and the loading rate in pN/s.

### Unbinding of SDC4 and fibronectin

Force pulling of an SDC4-exhibiting probe from a fibronectin-immobilized surface were conducted over a range of loading rates between 0.9 and 66 nN s^−1^. The distribution of rupture forces is shown in Fig. 3 *B*, and the dynamic force spectrum describing the unbinding is presented in Fig. 4 *B*. The energy landscape in this range is governed by single energy barrier and, when fitted with the Bell-Evans model in Eq. 1, is described by

(5) F=(12.2±0.6)lnr–(42±6),

with the rupture force in pN and the loading rate in pN/s. A correlation factor of R^2^ = 0.9844 was associated with this fit.

### Unbinding of decorin and fibronectin

DSMFS experiments using a decorin-exhibiting probe and a fibronectin-immobilized surface were conducted over a range of loading rates between 3 and 46 nN s^−1^. The distribution of rupture forces is shown in Fig. 3 *C*, and the dynamic force spectrum describing the unbinding is presented in Fig. 4 *C*. The energy landscape in this range is governed by a single energy barrier and, when fitted with the Bell-Evans model in Eq. 1, is described by

(6) F=(30.4±1.6)lnr–(215±15),

with the rupture force in pN and the loading rate in pN/s. A correlation factor of R^2^ = 0.9841 was associated with this fit.

The decorin-fibronectin curve, like that of SDC4 with fibronectin, cannot support a two-barrier model such as that observed for *α*_5_*β*_1_-integrin binding with fibronectin. In the case of the SDC4 interaction, the straight line crosses all data within error. The line misses only one datum for the decorin interaction. It can thus be concluded that a two-barrier model is inappropriate for these data.

#### Extraction of binding energies

From the linear Bell-Evans fits for each energy barrier revealed in the dynamic force spectra depicted in Fig. 4, energetic properties characterizing the binding interactions can be extracted. The width of each energy barrier, *χ*_B_, can be obtained directly from the gradient of the linear fit using Eq. 1. The dissociation rate describing each barrier, *K*_d_, can then be extracted from the intercept of the linear fit from Eq. 1. By incorporation of the Arrhenius relation (Eq. 2), an estimate of the thermodynamic energy of adhesion can be extracted from the dissociation rate directly or from the Bell-Evans fit by substitution. The parameters characterizing each barrier for the unbinding of SDC4, decorin, and *α*_5_*β*_1_-integrin from fibronectin are summarized in Table 1, noting that *K*_d_ and *ΔG* are calculated based on the assumption *f*_m_ = 10^7^ s^−1^.

**Table 1:** Extracted Energetics for Each Energy Barrier Revealed in the Dynamic Spectra for the Unbinding of Fibronectin from Both the Inner, I, and Outer, o, Barriers of *α*_5_*β*_1_-Integrin, as well as Those for SDC4 and Decorin

	*χ*_B_ (Å)	*K*_d_ (s^−1^)	*ΔG*/*k*_B_*T*
*α*_5_*β*_1_ (i)	0.69 ± 0.03	70 ± 40	11.9 ± 0.6
*α*_5_*β*_1_ (o)	6.6 ± 0.7	0.18 ± 0.13	17.8 ± 0.7
SDC4	3.4 ± 0.2	2.4 ± 0.4	15.2 ± 0.2
Decorin	1.4 ± 0.1	40 ± 4	12.4 ± 0.1

## Discussion

This study compares the interaction of the ECM protein, fibronectin, and the *α*_5_*β*_1_-integrin, with its binding of PGs. Analysis using DSMFS shows that in contrast to fibronectin-integrin binding, which is characterized by two distinct binding affinities, fibronectin association with PGs exhibits one binding site only. In addition, the results identify differences in elasticity between the three fibronectin interactions.

The dynamic force spectra extracted from unbinding measurements of both SDC4 and decorin with fibronectin revealed only a single energy barrier. This is similar to previous studies investigating the binding between heparin and fibronectin (32). Furthermore, the width of the energy barrier probed in this work for the SDC4 interaction with fibronectin is only ∼10% larger (and falls within the range of associated errors) than the width of 3.1 ± 0.1 Å reported for heparin (32). This would indicate a similar bond compliance in the binding of fibronectin, with both heparin and HS and its relatively large size suggests that the bond formed between PGs and extracellular fibronectin is resilient to mechanical stress and deformation. Combined, these results support other work showing that HSPGs bind fibronectin through their GAG chain and that the core protein has no significant role in binding (13).

Comparison of the dissociation rate extracted for the SDC4-fibronectin interaction in this study reveals a 12-fold increase when compared with that reported for the heparin-fibronectin (32), suggesting a weaker interaction between fibronectin and SDC4 than heparin. It can be noted that both the width of the barrier and the dissociation rate extracted from the dynamic force spectra for the SDC4-fibronectin interaction are comparable to values reported for other protein-carbohydrate interactions (53, 54).

The single energy barrier revealed in the dynamic force spectrum for the decorin-fibronectin interaction reveals a much smaller barrier width and a much larger dissociation rate than those extracted from the SDC4 interaction. The observed bond formed between decorin and fibronectin is therefore more brittle and less deformable than that with SDC4. Here, the binding strength between decorin and fibronectin is significantly smaller than that resulting from the association of heparin sulfate chains to fibronectin (3). However, under some conditions, affinities of heparan and chondroitin sulfates are comparable (55, 56).

Two energy barriers characterizing the unbinding of *α*_5_*β*_1_-integrin and fibronectin have been identified in this study. This is consistent with previous work on *α*_5_*β*_1_-integrin unbinding from fibronectin (30, 31) and with other studies of integrins with ECM components (57, 58). DSMFS data for the interaction of fibronectin with fibronectin-binding proteins in *Staphylococcus aureus* have also shown the possibility of two energy barriers (59), although this conclusion is not unequivocal. *S. aureus* binding to fibronectin is understood to involve *α*_5_*β*_1_-integrin (60). The interaction of fibronectin with monoclonal antibody has also been shown to exhibit two energy barriers (47).

Increasing loading rates reveal the internal binding regime, which is characterized by a large dissociation rate and a barrier of subångstrom width, indicating a brittle bond that is resistant to external forces. It is not uncommon to report subatomic internal barrier widths for ligand-receptor dissociation (30, 41, 57, 61, 62). Previous studies on the binding of the integrin *β*_1_ subunit with ECM proteins have suggested that this barrier is due to the ionic interaction between the RGD domain in cell-binding matrix components and the chelated Mg^2+^ ion in the *β*_1_ metal-ion-dependent adhesion site (58). The high-affinity barrier governs the unbinding at lower loading rates (and lower forces) and is characterized by a wide width, which implies that the bond in this regime can withstand significant deformation.

The slow dissociation rate for the outer barrier suggests a relatively high affinity bond between *α*_5_*β*_1_-integrin and fibronectin. This is consistent with the cell attachment function of integrin-ECM binding and suggests an integrin interaction through both the RGD and the synergy binding sites of fibronectin (30, 63, 64). Activation results in the change in conformation of the *β*_1_ subunit and permits higher affinity binding with ligands (65).

Other studies using DSMFS have reported that recombinant integrin variants exhibit binding characteristics of the high-affinity state, reflecting a conformational change in the protein (58). In contrast, the binding characteristics of the recombinant, PEG-linked *α*_5_*β*_1_-integrin variant (particularly the dissociation rate of the outer barrier) exhibited a lower affinity than that measured for the wild-type protein (30). It may be that *α*_5_*β*_1_-integrin requires an interaction with cellular components to induce structural stability in the receptor complex to provide efficient binding with extracellular fibronectin. It should be noted that both the dissociation rates and the width of each barrier extracted in this work are comparable to other integrin-ECM studies (30, 58, 66).

The estimated thermodynamic energy of adhesion for the outer barrier is within experimental uncertainty in agreement with the value of ∼17.3 *k*_B_*T*, which was extracted from equilibrium affinity constants acquired in surface plasmon resonance measurements for inactivated *α*_5_*β*_1_-integrin (67).

The characterization of the energy barriers involved in the dissociation of recombinant *α*_5_*β*_1_-integrin and fibronectin offers a good comparison and positive control for the unbinding energetics between fibronectin and the PGs SDC4 and decorin. This and earlier observations of two separate barriers for the *α*_5_*β*_1_-integrin interaction with fibronectin, compared with the single barriers revealed for the PG-fibronectin interactions, are also consistent with differences in the binding properties. The significant strength of binding between the cell-adhesion-mediating *α*_5_*β*_1_-integrin with fibronectin offers a useful comparison with the energetics of PG-fibronectin unbinding.

The lower affinity of the measured fibronectin binding of the PGs compared to that for *α*_5_*β*_1_-integrin may in part reflect PG interactions through the GAG chains and a stronger binding of the integrin through a protein-protein interaction (63, 68). This characteristic, as well as the two energy barriers in the dissociation mechanism, means that the bound complex of *α*_5_*β*_1_-integrin and fibronectin can sustain considerable deformation and is resilient to significant external forces.

In part, the distinct characteristics of the binding presented here reflect the differences in protein-protein and GAG-protein interactions with the integrin and PGs, respectively. These differences are also consistent with their specific molecular functions. The brittle interaction of decorin with fibronectin under low loading may provide increased sensitivity for regulation of matrix composition and signaling crosstalk (23, 69). In contrast, the ability to withstand deformation at focal adhesions and coordinate cell activation and movement may be facilitated by the greater elasticity provided by SDC4 and *α*_5_*β*_1_-integrin (3, 6).

## Conclusion

DSMFS was performed on the unbinding of fibronectin with SDC4, decorin, and *α*_5_*β*_1_-integrin. Single energy barriers were uncovered for both SDC4 and decorin unbinding with fibronectin, and two barriers were observed for the dissociation of *α*_5_*β*_1_-integrin with fibronectin. Using the Bell-Evans model, descriptive energetics of dissociation were extracted directly from the dynamic force spectrum characterizing each energy barrier.

The results identify differences in affinities and elasticities in PG and integrin binding to fibronectin, which are consistent with their distinct molecular characteristics and specific biological functions.

## Author Contributions

E.E.Q. conceptualized the problem. M.G. and Y.C. designed the AFM experiments. Y.L. and T.M.K. developed the chemical methodology. T.M.K. performed the AFM experiments and analyzed the data. M.G., E.E.Q., and T.M.K. wrote the manuscript to which all authors contributed. All authors reviewed and approved the manuscript before submission.
